# Targeted Therapies for Melanoma

**DOI:** 10.3390/cancers12092494

**Published:** 2020-09-03

**Authors:** Karel Smetana, Lukáš Lacina, Ondřej Kodet

**Affiliations:** 1 Institute of Anatomy, 1st Faculty of Medicine, Charles University, 128 00 Prague, Czech Republic; lukas.lacina@lf1.cuni.cz (L.L.); ondrej.kodet@lf1.cuni.cz (O.K.); 2 BIOCEV, 1st Faculty of Medicine, Charles University, 252 50 Vestec, Czech Republic; 3 Department of Dermatovenereology, 1st Faculty of Medicine, Charles University and General University Hospital, 128 08 Prague, Czech Republic

**Keywords:** melanoma, BRAF V600 mutation, advanced therapy of melanoma, therapeutic resistance

## Abstract

The incidence of cutaneous malignant melanoma is increasing worldwide. Despite available modern therapeutical options, long-term survival of patients in advanced stages of the disease remains rather limited until now. Detailed insights into etiopathogenesis and mechanisms of tumour progression enable physicians to manipulate distinct molecular structures and pathways therapeutically and so treat the tumour. Unfortunately, the acquisition of therapeutic resistance frequently terminates these therapeutical interventions. The presented special issue is focusing on the research and therapeutic experience of leading scientists, and it summarises the state of the art of targeted therapy of melanoma and suggests the new perspectives of the treatment of disease.

The incidence of cutaneous malignant melanoma (CMM) is significantly increasing worldwide. While surgical therapy of initial stages is straightforward and highly efficient, the treatment of advanced-stage disease remains problematic, making CMM the most deadly cutaneous malignancy. Moreover, melanoma is one of the leading cancers in average years of life lost per death from the disease [1]. Therefore, this important issue attracts attention, and melanoma urgently requires innovative therapeutic strategies, including targeted therapy [1].

In the previous decade, we have witnessed an obvious enthusiasm associated with novel promising therapeutics in clinics. By now, many of these drugs became first-line therapy options in our daily clinical routine practice. Some of them are even available for application in the adjuvant setting. However, we were also saddened by the fast appearance of old enemies. Primary and acquired treatment resistance, the plethora of adverse effects, treatment toxicity, the necessity of combination therapies, and unsustainable healthcare budget rise are just some examples of these renewed challenges.

For this Special Issue of *Cancers*, we have summoned 13 articles, including three reviews and ten original articles, submitted by the leading melanoma researchers. These articles address a variety of issues, ranging from the experimental in vitro studies to the results summarising experience from clinical trials and routine therapy of advanced stages of melanoma. The experimental studies bring detailed insight into the therapy resistance, which is vital for the refinement of the therapeutic indication.

It is widely known that activating BRAF mutations are present in approximately 50% of all melanomas. This high frequency of BRAF mutations was a starting point for a new chapter in targeted therapy for advanced melanoma. Later, mitogen-activated protein kinase (MAPK) inhibition in combination with the inhibition of BRAF and mitogen-activated protein kinase kinase (MAPKK) became available for the therapy of CMM with BRAF V600 mutation. Unfortunately, we are frequently dealing with a high proportion of patients with primary/secondary (acquired) resistance. Profiling of MAPK activity in clinical samples and cell lines can well distinguish between responders and non-responders, which can help to select patients for this type of therapy [2]. Pyridinyl imidazole compounds SB202190, SB203580 and SB590085 can be used to block the proliferation pathways in BRAF V600E melanoma cells. These molecules directly inhibit BRAF V600E kinase, and they also influence the lysosomes of cancer cells. Together, this may have a significant inhibitory impact on melanoma biology. The possible therapeutic effect of these compounds in clinics is promising [3]. The therapy of CMM with BRAF mutation is also addressed by Ruggiero and co-workers at the tissue culture level [4]. Their results demonstrate the role played by autocrine production of neuregulin in ErbB3 activity in the phosphorylation and AKT (protein kinase B) activation in the population of cancer cells, which can influence the biological properties of melanoma cells. Moreover, the activation of PI3K/AKT/mTOR (phosphatidylinositol-3-kinase/AKT/mammalian target of rapamycin) signalling plays a significant role in the mechanism of resistance to targeted therapy.

The expression of Raf kinase inhibitor protein (RKIP) is inversely associated with the aggressiveness of several types of cancers. Unfortunately, data about its role in CMM have so far been limited. Cristina Penas and co-workers [5] have clearly shown that RKIP is more extensively expressed in nevi than in CMM. Inverse association between RKIP expression and cell migration, differentiation and epithelial to mesenchymal interaction was also shown in CMM cells in vitro. RKIP detection in tumour samples can therefore be useful for diagnostics and refinement of the therapeutic indication. The therapeutic manipulation of RKIP can be used as a potential therapeutic modality in the future.

The KIT (receptor tyrosine kinase protein) oncogene plays a significant role in various aspects of melanoma biology. Somatic mutation in the c-KIT (tyrosine-protein kinase KITgene) represents only about 2–4% of somatic mutations in CMM in the Caucasian population (10% in Asians), but in mucosal and acral lentiginous melanoma, it is found in about 20% of cases. KIT inhibitors such as Nilotinib are efficient in the elimination of melanoma cells with mutation of this oncogene. Unfortunately, the KIT-mutated cells readily acquire resistance to inhibitors, with a positive contribution of FGF-2. So, the combination of KIT inhibition with the application of FGF receptor that immobilises FGF-2 could be a relevant approach in future therapy of CMM patients with mutated KIT [6].

In recent years, the stromal cells and mechanisms of the microenvironment have emerged as important factors in driving invasion and metastasis. Obviously, melanoma cells are not the only target within the tumour. Melanoma cells are components of a complicated tumour ecosystem in which the cancerous and noncancerous cells interact. The stroma represented by various non-malignant cells is not an innocent bystander and forms an indispensable functional unit that influences the progression of CMM [7]. The multiple facets of stromal biology in CMM and its role in potential induction of resistance to anticancer therapy is extensively summarised in the review by Diazzi and colleagues [8]. The presence of fibrotic stroma with cancer-associated fibroblasts in CMM seems to be one of the markers of poor prognosis in patients with advanced disease. The resistance to targeted therapy depends, in addition to other parameters, on the production of extracellular matrix as well as cytokines, including inflammation-supporting molecules, by the noncancerous cells of CMM stroma. Therefore, application of anti-fibrotic drugs such as Nintenadib or Sorafenib can be useful in the therapy of CMM with extensive fibrotic stroma.

The majority of articles in this Special Issue focus on various aspects of clinical application of the targeted therapy of CMM. The dabrafenib/trametinib (DAB/TRA) combination is used for therapy of BRAF-mutated patients. However, the clinical results exhibit a tremendous inter-individual variability between the treated patients. To adjust the therapeutic protocols and to improve the efficacy of anti-melanoma therapy, pharmacokinetic measurements of the therapeutics are desirable. This approach highlights the importance of individualised pharmacokinetic monitoring [9]. To monitor the efficiency of MAPK inhibitors, TERT (telomerase reverse transcriptase) promoter region mutations can be used as a biomarker. These mutations may impact the progression-free survival of patients with generalised CMM [10].

The therapeutic combination targeting mutated BRAF along with anti-PD1 therapy represents a highly efficient strategy in suitable patients. It offers a safe combination of improved survival, better therapeutic response and higher life quality of patients with generalised CMM [11].

Traditional chemotherapeutic agent Dacarbazine has been an important therapeutic option in melanoma treatment for many years. Until now, unresectable NRAS (N rat sarcoma)-mutated CMM represent a serious therapeutic challenge. The presented phase 2 clinical trial has demonstrated that MEK (mitogen-activated protein kinase kinase) 1/MEK2 inhibitor Pimasertib exhibits anticancer activity with a safety profile consistent with Dacarbazine [12].

In clinical practice, some common adverse effects can easily hamper all therapeutic efforts. MEK inhibitors significantly improve the efficacy of BRAF inhibition in comparison with BRAF inhibitor application only. Of note, some adverse effects, such as extensive vomiting and diarrhoea, were more frequently observed under combined therapy than in monotherapy [13].

A concise overview summarising the molecular background of brain metastasising of CMM and utilisation of these data in experimental therapy included in several clinical studies show new approaches to improving the clinical outcome of patients with metastatic central nervous system involvement [14].

A report from Germany summarising data on targeted therapy demonstrates the efficiency of this therapeutic approach in comparison with classical chemotherapy or adjuvant therapy using interferon. Of note, this study demonstrates that the efficiency of CMM therapy is region dependent. Surprisingly, a significant difference between the eastern and western regions in Germany was identified even a quarter of a century after the German reunion. Importantly, this indicates the profound impact of the economic level on patients suffering from the CMM and their survival [15]. CMM is a complex disease with multiple genetic alterations at the single-cell level under important epigenetic control of the cancer microenvironment. Individualised and closely targeted therapy of CMM must inevitably be based on the detailed molecular analysis of individual patients. Then and only then can it avoid the risk of therapeutic resistance acquisition and improve the survival rate and life quality of patients, as presented by authors of this Special Issue. However, collectively, prevention, public awareness, precise clinical diagnosis, and early surgical excision in an early stage of CMM still constitute the most efficient and cost-effective approach to CMM therapy with the greatest benefit for patients.
